# Supplementary material for: Are carbon emissions trading and green financial instruments synergistic? -Comprehensive quantitative research based on content analysis
Source: PLoS One. 2024 Mar 7;19(3):e0298601. doi: 10.1371/journal.pone.0298601 (PMC10919601; doi:10.1371/journal.pone.0298601)
Supplement: S1 Data — (ZIP) [file pone.0298601.s001.zip › Data/地级市Z_F工作报告.zip!/╣π╢1⁄2/╓Θ║ú╩╨/└┤╫╘╣1⁄2╓┌║┼í╛┬φ┐╦╩2╛▌═°í┐_2015.docx]

各位代表：

　　现在，我代表市人民政府，向大会作政府工作报告，请予审议，并请市政协委员和其他列席人员提出意见。

　　一、2014年工作回顾

　　过去一年，我们抢抓机遇、应对挑战，坚持稳中求进，全面深化改革，主动适应经济发展新常态，着力稳增长、调结构、惠民生，在全市人民的共同努力下，较好地完成了市八届人大四次会议确定的重点任务，实现了全年主要目标，经济社会发展取得了新成绩。

　　（一）沉稳应对经济下行压力，经济发展提速增效。

　　经济增长稳中有进。认真贯彻落实国家和省各项政策措施，抓住关键领域和薄弱环节，及时研究推出了培育大型骨干企业、促进外贸转型发展、壮大民营经济和总部经济等七个方面的政策和200多项具体举措，推动经济在结构调整优化中稳中有进、稳中提质，多项指标增幅在珠三角领先。全年实现地区生产总值1857.3亿元，增长10.3%。人均GDP预计超过1.8万美元。一般公共预算收入224.3亿元，增长23.6%。GDP和预算收入增速均居珠三角第一。一般公共预算收入占GDP的比重达12%，税收收入占一般公共预算收入的比重达81.2%。规模以上工业企业利润总额达293.6亿元，增长18.2%。全体居民人均可支配收入初步测算达33234.9元，增长9.5%。全市本外币存贷款余额同比分别增长10.9%和17.1%。城镇登记失业率为2.26%。居民消费价格上涨3.1%。发展的质量和效益继续提升。投资保持较快增长。以加快基础设施现代化为战略重点，投资力度持续不减，固定资产投资首次突破千亿元，达到1135.1亿元，增长23.5%，增速连续四年保持珠三角第一。完成重点建设项目投资512.8亿元，为年度计划的132.5%。一批内通外联的交通枢纽工程全面启动。港珠澳大桥主桥及珠澳人工岛建设顺利，珠海连接线配套工程进度过半。金琴高速、横琴二桥等42个项目进入全面施工期。金湾互通立交、梅华路改造等13个项目建成。珠海北站TOD综合开发、城轨延长线、15万吨级主航道工程、南屏大桥等28个项目开工。投资结构进一步优化，基础设施投资、工业投资和服务业投资分别达355.7亿元、276亿元和857.3亿元，占全社会投资比重分别为31.3%、24.3%和75.5%。完成工业技术改造投资58.3亿元，增长156.7%，增速居珠三角首位。民间投资增长55.7%，占比达34.8%。

　　消费出口平稳。积极组织开展各类消费促进活动，实现社会消费品零售总额815.7亿元，增长13.2%。着力培育旅游、会展消费新热点，全市旅游总收入261.8亿元，增长8.3%。珠海国际会展中心、华发商都等项目运营，第十届中国航展观众和签约项目再创历史新高，长隆国际海洋度假区首期建成，接待游客超过800万人次。海洋海岛游、乡村生态游蓬勃发展，竹洲水乡水利风景区、莲洲镇分别被评为国家级水利风景区和广东省休闲农业与乡村旅游示范镇。大力催生新兴业态、新商业模式和新消费增长点，信息消费增长20%，电子商务交易额增长15.8%。推动外贸在转型中稳定增长，进出口总额达550亿美元，增长1.3%，其中出口增长9.3%。一般贸易进出口占比提高到53.5%，内资企业出口占比达55.2%，加工贸易委托设计和自主品牌混合生产方式出口占比达62%，服务外包离岸执行金额增长37%，外贸结构进一步优化。保税区进口商品交易中心跨进全省首批十大进口商品交易中心培育行列，新增首家省级外贸综合服务平台企业。新批外商直接投资项目330个，新引进投资超过千万美元项目39个，实际吸收外资19.3亿美元，增长14.4%。

　　各类企业健康发展。实行培育大型骨干企业、服务中小微和民营企业各项政策措施，促进了各类企业竞相发展。财政金融扶持实体经济力度加大，全年为企业减免各类收费4000余万元，新增贷款354.3亿元，社会融资规模达632.5亿元。新增年主营业务收入超百亿元以上企业2家，总数达到6家，格力电器销售收入超过1400亿元。新增上市公司1家，总数达32家，其中24家境内上市企业市值超过2700亿元。民营企业工业增加值增长10.6%，民营经济增加值占GDP比重达32.3%，对经济增长贡献率达23.9%。

　　（二）深入实施创新驱动发展战略，产业转型升级步伐加快。

　　自主创新取得新进展。启动创新型城市建设三年行动计划。全社会研发投入占GDP比重达2.6%，每百万人口发明专利申请量达到1763件，均位居全省第二。实施了扶持新型科技研发机构政策，新建了一批新型研发机构，中美食品安全研究中心和世界食品中心、珠海诺贝尔国际生物医药研究院、清华珠海创新中心、华南理工大学珠海现代产业创新研究院等新型研发机构落户。落实企业研发费税前加计扣除等鼓励政策，减免企业所得税额2.8亿元。格力电器“基于掌握核心科技的自主创新工程体系建设项目”荣获国家科技进步二等奖。丽珠集团原创新药获得广东省科技进步一等奖。投入专项资金招才引智，新增留学人才创业项目42个、省创新创业团队1个、省领军人才2名、“国家千人计划”特聘专家6名，创新人才队伍加快发展。实行了孵化器倍增计划，制定了促进科技金融融合发展政策措施，一批科技金融机构投入运营，新增股权（创业）投资企业192家、新三板挂牌企业12家。国家知识产权运营公共服务试点平台落户横琴。全面加强标准化、知识产权、品牌和计量基础工作，创建全国质量强市示范城市取得新成绩。

　　产业升级步伐不断加快。实施了先进装备制造业、会展业、金融业等专项发展规划和产业政策，大力推进重大产业项目建设，促进了“三高一特”产业体系发展和产业结构优化升级。全市三次产业比例进一步优化为2.6：50.6：46.8。二次产业优先发展，规模以上工业增加值897.9亿元，增长11.2%。先进装备制造业不断壮大，海洋工程装备制造、轨道交通、航空、新能源汽车等产业形成新特色。新引进和在建装备制造项目95个、总投资1370多亿元，中海油系列项目、中国北车珠海基地、瓦锡兰玉柴中速机等一批大项目建成投产。先进制造业、战略性新兴产业增加值分别增长20.1%和11.5%，占规模以上工业增加值的比重分别达到47.3%和15%。现代服务业集聚发展，服务业增加值增长8.7%，现代服务业增加值占比提升至57.6%。金融、会展、总部经济等产业培育取得新进展，金融业增加值增长11.5%，新兴金融机构总数达267家，增长196.7%。新引进亚洲通用航空展、中国（珠海）国际海洋高新科技展览会、中国（珠海）国际智能电网展览会等国际性品牌项目，会展城市影响力提升。新认定总部企业36家，贡献地方税收近13亿元，一批央企和民企实业型总部企业植根发展。高新技术产业规模递增，高新技术产品产值占规模以上工业总产值预计达54.7%，提高了1个百分点，占比居全省第三。新增国家高新技术企业40家、火炬计划重点高新技术企业5家，国家高新技术企业总数达346家。涌现出云洲智能、健帆生物等新一批创新型企业。移动互联网产业增长75.6%，软件企业营业收入增长20%，智能电网行业产值增长35.8%。小米通讯、魅族通讯销售额分别增长了1倍多和2倍多。生态农业和海洋经济加快发展，农林牧渔业增加值增长3.9%，新发展农民专业合作社50家，引进大型特色农业企业7家；海洋产业产值达850亿元，增长20.7%，海岛保护和开发取得新进展。

　　（三）加快重大产业集聚区发展，多极增长新动力不断增强。

　　横琴新区实现五年成规模目标。新区创新改革增创了新优势，自贸试验区获批，所得税优惠政策落地实施，横琴口岸实现24小时通关，二线通道封关运作，自贸区和新区的政策叠加优势凸显。现代服务业加速崛起，总投资超过2500亿元的65个重点项目进展顺利。金融创新取得新突破，金融企业发展到670多家，实现境外融资29.5亿元，跨境人民币同业存款业务总额达470多亿元。大横琴公司在香港发行内地首只地方城投债15亿元。广东金融资产交易中心交易额超过1700亿元。大力配合澳门建设“一中心、一平台”，出台了横琴支持澳门经济适度多元发展措施，319家港澳企业落户横琴，投资总额达2220多亿元，对澳项目供地占总建设用地比重达53%。粤澳合作产业园、粤澳合作中医药科技产业园建设加快，珠港澳融合发展呈现新局面。岛内现代化基础设施配套进一步完善，路网、管网、水网、电网、信息网“五网”工程基本建成投入使用。

　　重点区域发展齐头并进。高栏港加快建设第三代港口，吞吐能力达1.5亿吨，港口货物吞吐量增长6.7%，疏港铁路一期工程通车运营，16个临港产业项目竣工投产，在建筹建重大项目42个、投资近1000亿元，装备制造业龙头作用和经济腾飞引擎功能增强。高新区成为科技创新的“领头羊”，新引进移动互联网、生物医药器械等高新技术企业和项目55个，投资额达54.4亿元，规模以上高技术制造业增加值增长29.3%。航空产业园已落户项目投资超过170亿元，基础设施和园区配套工程加快推进，首款具有自主知识产权全复合材料涡桨公务机“领世AG300”成功首飞，国内首条“珠海-阳江-罗定”的低空航线开通，机场客运量超过400万人次，增长近40%。富山工业园成功创建省绿色升级示范工业园，惠普智慧产业园、修正国际医药健康产业城等一批重点项目加紧推进，市政道路等配套工程建设提速。各功能区域相互支撑、协调联动的发展新格局加快形成。

　　（四）全面深化改革扎实推进，对内对外开放进一步扩大。

　　重点领域改革取得新突破。66项年度重点改革任务基本完成。大力推进政府职能转变和简政放权，完成新一轮政府机构改革，调整优化了卫生计生、食品药品监管等10个政府部门的主要职能。公布了39个政府部门的“权力清单”，制定了投资及相关领域的“负面清单”。动态调整行政审批事项两批次12项，建立了市、区、镇街、村居四级行政审批事项和社会服务事项目录，镇街行政服务中心和村居公共服务站实现全覆盖，行政审批和社会服务事项网上办理率达到100%。建立“一条例、一办法、七制度”的商事登记法规新体系，催生了投资创业热潮，全市每千人商事主体数量突破120个。开展了横琴新区探索综合行政执法试点。实行了新的财政管理体制，市直部门预决算在网上公开。制定实施国资国企改革创新政策，有序发展混合所有制经济，实行公用事业领域特许经营权改革。推进农村综合配套改革，开展农村土地承包经营权确权登记，“三资”监管交易平台覆盖全市村居，斗门区被确定为第二批中央农办农村改革试验联系点。推进集体建设用地使用权流转、不动产统一登记、陆海统筹试点。优化国有文化资产监管机制，组建珠海报业、广播影视两个传媒集团。稳步推进医改，我市被列为公立医院改革国家联系试点城市，研究制定了药品和医用耗材零差率等改革政策。开展构建社会组织综合监管体制试点。深化生态文明制度创新，开展了划定生态红线、生态补偿等改革。

　　对外开放合作向纵深拓展。开拓对外开放新领域，落实国家“一路一带”战略，积极参与21世纪海上丝绸之路建设。全市对外投资项目45个，协议中方投资金额7.2亿美元，同比增长293%。成功举办第十一届世界家庭峰会和第六届潮商大会，扩大了国际交流空间。完善了珠港、珠澳联席会议制度，合作机制进一步加强。全面实现珠港澳服务贸易自由化，新增港澳企业289家，利用港澳资本13亿美元。珠海驻港澳中资企业资产总额达200多亿港币。加快跨境基础设施对接，珠澳城轨对接工程开工，粤澳新通道等项目稳步推进。推进通关便利化改革创新，拱北口岸实施延关，珠澳跨境工业区口岸临时扩大开放，拱北海关推广了“先进后报”监管模式。扎实推进珠中江经济圈建设，累计签订64项合作协议，29个重点合作项目加快实施。统筹做好对口帮扶工作，落实阳江、茂名两市帮扶村项目777个、新建产业基地32个、建成基础设施类项目758个，带动贫困村集体收入和贫困户人均收入分别增长了8倍和2.9倍。实施重庆巫山、四川凉山扶贫协作项目38个。启动市内欠发达村帮扶项目214个。

　　（五）大力建设国际宜居城市，生态家园更加美好靓丽。

　　生态文明建设取得新成效。深入实施生态文明建设促进条例和建设国际宜居城市决定，通过国家生态市现场考核验收和国家园林城市复查，各级生态示范区（镇、村）覆盖率达到80%以上。划定生态控制线，确保受保护生态用地不少于1000平方公里，绿色开敞空间占国土面积比例不低于70%。完成省林业重点生态工程建设任务，新建碳汇林3.2万亩、生态景观林带96.7公里，新建和提升森林公园21个，新增绿道105公里，人均公园绿地面积达到18平方米，森林覆盖率达33.6%。有效开展裸露山体整治复绿工作。污水管网建设全面铺开，前山河流域环境整治完成旧村截污一期工程，生活垃圾处理生态环保工厂建成。工业、建筑、交通、公共机构等重点领域节能减排扎实推进，单位工业增加值能耗下降8.49%，二氧化硫排放量和化学需氧量排放量预计分别下降7.1%和3%。淘汰“黄标车”2.3万余辆，完成年度任务的116%。绿色建筑建设规模、标识数量居全省前列。全市PM2.5浓度下降10.5%，环境空气质量位居全国重点城市前列。珠海在《2013年城市可持续发展指数报告》中名列前茅。

　　城市优化发展取得新进展。完成城市概念性空间发展规划编制和城市总体规划修改，全面推进“五规”融合，新型城镇化规划体系进一步完善。统筹平衡、科学规划新城新区建设和老旧城区改造，进一步优化城市格局，着力提升城市的功能品质和特色化水平。全面展开西部生态新区起步区、平沙新城、金湾航空新城、富山产业新城、唐家科教新城的土地开发和骨干路网等基础设施建设。加快推进14个城中旧村更新项目和61个城市更新项目，完成232个老旧小区基础设施和“四防”整治提升，改造了83家农贸市场。海滨泳场完成升级改造，成为珠三角独具风情的美丽海湾。推进“三清”、“两违”整治行动，共清理土地11900多宗、项目近2700个，清理政策涉及的项目170多个，整治违法用地3800多亩、违法建筑400多万平方米，有效提升了城市发展空间和环境品质。加强城市交通拥堵治理，加快城市公共交通发展，有轨电车1号线首期工程实现联调联试，新投放一批新能源公交车和出租车。建成一批人行过街通道和东部城区慢行系统。

　　智慧城市建设取得新成果。基本建成区域医疗“一卡通”、智慧交通等重点项目。完成智慧城市云计算平台规划。市民个人网页和企业专属网页用户分别超过60万和14.4万。12345综合服务热线增加了微信和网站服务新功能。“政民通”系统覆盖94个社区。加快推进信息基础设施建设，新建3G/4G基站8200多座、WIFI热点近7000个，完成光纤接入用户超过20万户，光纤入户率超过41%，居全省第二。

　　幸福村居创建取得新突破。深入实施幸福村居“六大工程”，209个村居实现规划全覆盖，200人以上自然村实现公路硬底化，新建成100个村居污水处理点，新增8个农村垃圾分类试点，209个村居告别“垃圾围城”，30个村居被评为省级卫生村，乾务镇、南屏镇被评为省卫生镇，斗门南门村被评为“中国十大最美乡村”。 农村传统农业加快向高附加值的都市农业、观光休闲农业发展转型，合作社组织能力进一步增强，人居环境和生产条件持续改善。

　　（六）努力增进民生福祉，人民生活水平不断提高。

　　民生和社会事业投入力度加大。把做好民生工作的立足点放在守底线、保基本、补短板、促均衡上，推进基本公共服务均等化，创建社会建设示范市。全市一般公共预算九项民生支出172亿元，增长13.8%，占比提高到63.6%。扎实办好十件民生实事，一批事关群众利益的就业、教育、医疗、住房等热点难点问题得到解决。

　　教育、文化、卫生事业加快发展。扎实推进教育创强争先，三个行政区成功创建省推进教育现代化先进区和义务教育均衡区，金湾区和斗门区还通过了国家义务教育均衡区评估验收。小学的免费义务教育生均公用经费补助标准提高到950元，初中的提高到1550元。规范化幼儿园比例达到86.9%，异地务工人员随迁子女入读公办学校比例达到67.3%。“一院两馆”主体工程基本完成。免费开放市文化馆新馆，新建成28个社区文体公园和100个村居文化中心，50%以上村居建成了数字农家书屋，一指禅推拿项目入选第四批国家非物质文化遗产。成功举办首届市民文化节、国际情侣双人自行车赛等文体活动。新建成一批医疗卫生基础设施，就医环境不断优化。50多个家庭医生团队与超过19万户家庭签订了服务协议。人口均衡型社会示范市建设扎实推进。已有2900多对夫妇享受“单独两孩”政策。有效防控了登革热、埃博拉等疫情。

　　就业和社会保障水平持续提高。城镇新增就业4.6万人，开展就业培训16万人次。新建3家市级创业孵化基地，新增创业人数2700多人。率先建立统一的城乡居民基本养老保险制度，惠及25.5万城乡居民，退休人员平均养老金增长10%。率先实现城乡低保标准一体化，城乡月人均低保补差水平、农村五保年集中供养和分散供养标准、城乡居民医疗保险财政补贴标准不断提高，保持在全省领先水平。补充医疗保险支付待遇4000多万元，共7200多人次受惠，进一步减轻了群众大病医疗负担。与周边城市30家医院实现异地就医即时结算。实施困难群众医疗救助2.1万多人次，投入1300多万元。健全特殊群体服务保障制度，发放残疾人生活津贴2400多万元。完善养老服务体系，探索社会养老服务多元化发展方式，居家养老服务覆盖城镇社区和90%的农村社区。完成省下达的保障性住房建设任务，基本建成3895套，新开工3522套，推出公共租赁住房2300套。完成天然气入户置换，惠及全市12.3万户居民。

　　社会保持和谐稳定。“平安珠海”十大工程重点项目有序推进，视频监控系统（二期）全面建成，“三联村居”建设覆盖70%村居。推动建立党政统筹的社会创安长效机制，强化立体化社会治安防控体系，率先在全国发布镇街级平安指数，重拳打击严重违法犯罪活动，大力整治治安突出问题和重点地区，全市接报违法犯罪警情同比下降6.2%，群体性事件下降42%，群众安全感和满意度提升。创新社会矛盾预防化解机制，排查重点矛盾纠纷化解率达98.8%。建立健全劳资纠纷预防机制，制定建设工程领域保证金、预储金和从业人员实名制管理办法，实行企业欠薪应急周转金管理，开展农民工工资支付情况专项检查，为2.8万余名劳动者追回工资1.7亿多元。深入创建全国安全示范市，应急管理和预防处置突发事件能力不断提高，安全生产形势总体稳定，生产安全事故宗数下降6%。强化食品药品监管，加大对假冒伪劣、虚假广告专项整治力度，确保人民群众吃得放心、用得安心。双拥共建深入开展。国家安全、国防建设和民兵预备役工作继续加强。司法行政取得新成绩。海防打私力度加大。外事交流合作进一步扩大。民族宗教、人防、侨务等其他工作都取得了新进步。全市动员、全民共创全国文明城市，顺利通过全国文明城市考评，城市文明发展水平迈上了新台阶。

　　过去的一年，我们始终坚持全面推进依法行政，加快建设法治政府，取得了可喜成绩，在《中国法治政府评估报告2014》中位列第十。政府立法制度和程序不断完善，提请市人大常委会审议法规草案5件，制定政府规章7件，法治保障进一步加强。科学民主决策水平不断提高，完善了重大行政决策合法性审查、专家咨询论证和听证制度，举行重大行政决策听证会13场。网络问政深入开展，政务公开进一步深化。行政执法监督加强，办理行政复议案件282件。以改进政风推进廉政建设，扎实开展群众路线教育实践活动。严格落实“八项规定”，全面完成79项整改任务，“三公”经费下降32.3%，政府作风加快转变，政务服务水平不断提高。加大监察、审计力度，强化执纪问责，严肃查处违纪违法案件，全市250多名公职人员受到了党纪政纪法纪处理。坚持定期向市人大及其常委会报告工作，向市政协通报情况。认真办好议案决议和建议提案，全年办理建议提案663件，已办结污水和垃圾处理设施建设议案，斗门四小联围海堤达标建设任务完成过半，市技工学校改扩建工程进入实质性建设阶段。

　　各位代表！过去一年的成绩来之不易、催人奋进，这是全市人民同心同德、艰苦努力的结果。在这里，我代表市政府，向各位代表、政协委员和全市人民，表示衷心的感谢！

　　我们也清醒认识到，经济社会发展长期积累的经济结构不尽合理、自主创新能力不强、区域发展不协调、基础设施承载力不足、资源环境约束加大等深层次问题和矛盾依然存在。当前经济运行中，投资增长接续能力受限、基础设施投资增长难度加大、制造业投资不足；消费需求增长偏弱、新的消费热点尚未形成；外贸转型升级亟待加速；重大产业项目建设周期和发挥效能时间长，企业生产经营困难增多，巩固经济持续向好势头的压力较大；转变政府职能、改善民生、城市精细化管理等任务依然繁重，这些问题和困难仍然突出，全面深化改革和转方式、调结构的任务十分艰巨。我们要正视这些问题，采取有力措施加以解决。

　　二、2015年工作部署

　　当前，我国经济发展进入了一个新常态，在增长速度、发展方式、经济结构、发展动力等方面呈现一系列新的趋势性变化，正在向形态更高级、分工更复杂、结构更合理的阶段演进。总体来看，今年我市面临的形势仍然复杂多变，全球经济总体复苏疲弱，国内经济处于增速换挡期、结构调整阵痛期、前期刺激政策消化期“三期叠加”阶段，存在不少不稳定不确定因素，经济下行压力将贯穿全年，潜在风险增加。同时也要看到，全国经济形势总体向好的基本面没有改变，仍然处于大有作为的重要战略机遇期。对珠海而言，今后一个时期支撑发展的有利因素更多。持续有力的投资使基础设施承载力不断增强，珠江口西岸交通枢纽地位已经确立，港珠澳大桥时代正在到来；重大工程和重点区域建设促进“三高一特”产业形成发展，为稳增长、调结构提供了有力支撑；横琴开发和自贸试验区建设使珠海站在新一轮改革开放的前沿，在制度创新上保持先发优势，与港澳融合发展不断深化，珠海有基础、有条件在新常态下继续保持较高的经济增长速度，实现更有质量、有效益、可持续的发展。我们必须全面、科学认识当前形势，既要增强忧患意识，把防范风险、应对困难特别是应对经济下行压力的工作做得更加扎实充分，更要牢牢把握机遇，坚定战略自信，保持战略定力，努力推动珠海加快适应新常态、引领新发展、再上新台阶。

　　——适应新常态，就是要准确把握经济新常态的基本特质和珠海发展的阶段性特征，主动顺应发展新趋势，开拓发展新征程。必须坚持建设生态文明新特区、科学发展示范市和珠江口西岸核心城市的定位不动摇，以生态文明理念统领经济社会发展，坚定不移地扭住转方式调结构主线，找准速度和质量的平衡点，努力在保持经济较快增长中实现产业中高端发展。必须更加注重创新驱动发展，坚持把创新驱动作为核心战略来抓。必须更加突出发展质量效益，始终坚持以经济发展质量和效益为中心，增强珠海经济核心竞争力。

　　——引领新发展，就是要紧抓当前发展机遇和良好势头，在国家战略中把握新高度，树立新标杆。要把握好建设“21世纪海上丝绸之路”和广东自贸试验区两大战略新机遇，以横琴建设推进体制机制和制度改革创新，打造自贸试验区建设新典范，始终引领改革开放新潮流。要积极迎接港珠澳大桥时代，全面建成珠江口西岸交通枢纽，高起点参与“走出去”区域开放合作，打造“21世纪海上丝绸之路”重要节点，创立国际竞争的新维度。要大力促进珠港澳更紧密融合发展，打造珠港澳国际都会区，树立国际宜居城市建设新示范。要协同共建珠江西岸先进装备制造产业带，打造先进装备制造业龙头，建设珠江口西岸产业转型升级新高地。

　　——再上新台阶，就是要以建设珠江口西岸核心城市为方向，加快向更高的发展层次跃升。要努力实现经济总量在珠三角争先进位，人均水平保持领先，产业结构进一步优化升级，社会建设水平位居全国前列，民生福祉全面提升，改革创新活力进一步激发，社会公平正义充分彰显，成为广东发展新的增长极、科学发展的排头兵。

　　今年政府工作的总体要求是：全面贯彻落实中央、省和市委的各项决策部署，以全面深化改革增创发展新优势，以推进创新驱动重塑增长新动力，以建设横琴自贸试验区树立开放新标杆，以发展珠江西岸先进装备制造产业带打造产业新高地，以打造法治政府形成法治新保障，不断提高发展绩效，持续增进民生福祉，实现经济持续健康发展和社会和谐稳定，推动生态文明新特区、科学发展示范市建设迈出新步伐。

　　建议今年经济社会发展的主要指标是：地区生产总值增长10%；规模以上工业增加值增长11%；社会消费品零售总额增长12%，固定资产投资增长15%，外贸进出口保持较好增长；一般公共预算收入增长12%；城乡居民收入增长与经济增长基本同步；居民消费价格涨幅和城镇登记失业率分别控制在3%和3.2%以内；节能减排完成省下达的任务。

　　今年的重点任务和主要工作是：

　　（一）坚定不移全面深化改革开放。

　　改革开放是新常态下引领发展的最大动力。要落实好各项改革攻坚任务，加快构建开放型经济新体制，全面释放内生动力和发展活力。

　　发挥经济体制改革的牵引作用。执行新一轮财政管理体制，科学合理划分各级政府事权和支出责任。贯彻新预算法，改善预算管理。建立规范的政府举债融资机制，探索创新政府与社会资本合作模式。规范发展私募投资基金，推进小微金融改革创新，大力发展互联网金融，支持设立和发展金融租赁、消费金融、合资证券公司、民营银行等法人金融机构。“一企一策”推动国资国企规范、有序改革，发展混合所有制经济。加快国有资产优化重组，提高国有资本证券化率。建立“大国资”监管体系。完善法人治理机构，探索建立职业经理人制度，健全激励约束分配机制。深化国土资源管理改革和农村综合改革。全面完成农村集体建设用地（宅基地）和留用地使用权确权登记工作。稳妥推进农村土地征收、集体经营性建设用地入市和宅基地制度改革。推进农村金融改革和股份制改革。

　　深化行政审批制度改革。加快建成行政审批项目最少、行政效率最高、行政成本最低、行政过程最透明的地区。继续取消调整一批审批事项，完成非行政许可审批事项清理工作。推进行政审批标准化建设。执行政府“权力清单”制度，公布各区“权力清单”，编制市区部门纵向“权力清单”。进一步探索和完善法定机构试点。完善四级政务服务体系，拓展网上办事大厅功能，建设电子证照库。推广企业专属网页和市民个人网页。完善商事制度改革后续监管配套措施，实行“三证合一”登记制度。深化社会信用体系和市场监管体系建设，加快社会信用信息平台和市场监管信息平台互联互通。推行企业投资项目清单管理。

　　深化社会治理体制改革。加快创建政社分开、权责明确、依法自治的现代社会组织体制，注重运用法治方式，实行多元主体共同治理。以建设人口均衡型社会为目标，有序推进户籍制度改革。深入推进社区行政事务准入。探索建立区、镇（街）外来人口服务管理中心。推进“法律顾问进村居”。培育和发展公益服务类社会组织，充分发挥社会组织培育发展中心的作用。进一步提升社会工作队伍的专业化水平。

　　加快构建开放型经济新体系。全面参与“21世纪海上丝绸之路”建设。推动巴基斯坦瓜达尔合作项目。落实与贵州黔南等沿铁区域陆港物流园合作。积极配合澳门建设世界休闲旅游中心和与葡语系国家国际贸易平台。加强珠港澳旅游业、会展业合作。发展保税展示贸易，建设航空器材标准件和文化艺术品等保税交易中心。发挥万山港口岸“一关三区”的独特作用，推进万山海洋科技示范园规划建设。深入实施创新招商引资三年行动计划，开展定向招商、产业链招商，实际吸收外商直接投资增长5%。建立内地企业“走出去”综合服务平台和保障体系，支持企业开展跨国并购、海外股权投资，建设境外营销网络和产业园区。加强对外联系交流，举办第二届世界广府人恳亲大会等国际性活动。继续深化珠中江及粤西区域合作，全面落实69个珠中江重点合作项目。推进对口凉山州等地区扶贫协作，全面完成阳江、茂名对口帮扶和扶贫“双到”任务。

　　（二）大力实施创新驱动发展战略。

　　坚持把创新驱动作为核心战略，加快完善区域创新体系，整体提升自主创新能力。

　　强化企业为主体的自主创新体系。制定新型研发机构发展三年行动计划，支持企业建设新型研发机构，创建产业技术创新联盟和创新创业园。支持国家船舶和海洋工程装备材料质检中心、珠海诺贝尔国际生物医药研究院、清华珠海创新中心、华南理工大学珠海现代产业创新研究院等载体建设。鼓励企业和高校、科研院所开展协同创新，在先进装备制造、战略性新兴产业领域开展共性技术和关键核心技术攻关，推进专利和科技成果标准化、产业化。深入实施各类人才计划，加大领军人才与创新团队引进培育力度，为技术创新提供智力保障。推进国家知识产权试点城市建设，提高知识产权创造、运用、管理和保护的能力。建设全国知识产权运营公共服务试点平台。全面开展全国质量强市示范城市创建工作，构建现代质量监管模式。

　　推动科技、金融和产业融合发展。健全科技金融服务体系和配套政策。搭建金融服务平台和多层次创业投融资体系。推动设立科技支行和政府控股或参股的融资性担保机构。规范发展股权（创业）投资机构，发展天使投资和风险投资，引导社会资金支持科技创新。推进金融产品创新。落实科技企业上市扶持政策。加快发展科技服务业。

　　推进信息化建设和应用。完善新一代信息基础设施，加快建设智慧城市应用项目，推进城市基础设施智能化。推动信息技术应用、产业发展与工业转型升级融合互动、协调发展。发展信息技术产业，构建云计算、大数据和物联网等应用创新平台。推广应用物联网、云计算、大数据等新兴技术与各行业融合。发展工业机器人、自动化生产线等智能装备，推动工业生产向网络化、智能化和服务化转变。支持企业利用电子商务创新营销模式、优化业务流程。

　　发挥高新区创新龙头作用。打造实施创新驱动发展战略主战场，促进项目、资金、人才、政策等创新要素加速集聚。大力培育创新文化。全方位推动产品创新、品牌创新、产业组织创新和商业模式创新。建设科技金融创新示范区。完善软件与集成电路、移动互联网、智能电网、医疗器械等产业规划。落实孵化器倍增计划，打造覆盖研发、孵化、平台、生产的完整产业链。推进智能产业园、金山软件园、珠海信息港、清华科技园二期等项目建设。

　　（三）突出抓好横琴自贸试验区建设。

　　自贸试验区建设是新时期珠海全面深化改革和扩大开放、促进与港澳深度融合发展的重大机遇。要以制度创新为核心，对接国家重大战略，坚持科学开发、从容建设，将横琴自贸试验区建设成为新一轮改革开放先行地、粤港澳深度合作示范区、“21世纪海上丝绸之路”的重要节点，为全面深化改革和扩大开放探索新途径、积累新经验。

　　建设法治化国际化营商环境。在扩大开放、体制机制改革等方面的法制建设上先行先试，加快形成高标准投资贸易规则体系。推动制定自贸试验区条例，完善法制保障体系。强化自贸试验区与港澳服务贸易自由化和投资便利化制度建设，加快制定一批实施细则和操作指南。发展国际仲裁、商事调解机制，完善自贸试验区国际法律服务体系。率先创新出入境管理机制和口岸监管模式，探索实施“合作查验、一次放行”和“两地一检”等新模式，积极配合国家开展查验机制创新试点工作。建立行政权力清单制度。推进行政审批标准化、信息化建设。推进综合执法改革试点工作，建立集中统一的综合行政执法体系。实行统一的市场准入制度，建设市场准入统一平台。对外商投资实行准入前国民待遇加负面清单管理模式，对负面清单之外的领域，将项目核准和企业合同、章程审批改为备案制。实行企业投资备案项目自动获准制。落实企业违法责任提示清单制度。

　　深入推进粤港澳服务贸易自由化。促进澳门经济适度多元化，进一步扩大对港澳服务业开放，进一步取消或放宽对港澳投资者的资质要求、股比限制、经营范围等准入限制，重点在金融服务、商贸服务、专业服务、科技文化服务、社会公共服务和休闲度假等领域取得突破。加强与港澳金融、商贸、专业服务等生产性服务业合作。与澳门共同编制粤澳合作产业园产业规划，建设港澳现代服务业聚集发展区。发展高端医疗服务。加快建设粤澳合作中医药科技产业园，加快推进国家食品安全（横琴）创新工程。

　　促进服务要素便捷流动。对高层次人才在出入境、签证居留、项目申报、创新创业、评价激励、服务保障等方面给予特殊政策。推进粤港澳服务业人员职业资格互认。探索在横琴工作、居住的港澳人士社会保障与港澳有效衔接。加快实施澳门车辆在横琴与澳门间便利进出政策。

　　建设粤港澳金融创新改革试验区。推动横琴银行开展离岸金融业务，继续开展跨境人民币双向融资试点，促进跨境保险等金融创新尽快开展实际业务。降低金融机构准入门槛，金融服务业对港澳地区进一步开放，建立与粤港澳商贸、旅游、物流、信息等服务贸易自由化相适应的金融服务体系。发展面向港澳和国际的新型要素交易平台。

　　（四）努力保持经济稳定增长。

　　加快建设先进装备制造基地。以打造珠江西岸先进装备制造业发展龙头和国内领先、具备国际竞争力的装备制造集聚区为目标，以高栏港区为中心，航空产业园、富山工业园为两翼，以重大项目为抓手，全面实施先进装备制造业发展规划和三年行动计划，加快建设船舶与海洋工程、航空航天、轨道交通、新能源汽车、高端电子电气、智能制造等六大产业基地，提升产业规模和质量。今年力争实现装备制造业总产值1900亿元，增长18%。强化项目服务保障，突出抓好47个去年签约项目和48个在建项目建设，推动中联重科、新兴际华、格力商用空调等项目开工建设，促成三一海洋重工产业园一期等项目建成投产，促进中海油系列项目、中航通飞等近年来新投产项目尽快达产。实行精确招商，着力引进国内外大型龙头骨干企业和拥有核心部件制造技术的配套企业，培育若干个在全国全球有影响力和竞争力的好项目。办好首届珠江西岸先进装备制造业投资贸易洽谈会、亚洲通用航空展等展会，提高产业集聚度。启动高栏港装备制造荷包基地用海申报工作，高起点加快完善航空产业园和富山工业园基础设施配套，提升“一区两园”综合承载力。深化“六市一区”产业协作，推进珠江西岸先进装备制造产业带建设。

　　保持投资合理较快增长。继续发挥投资的关键作用，保持投资增长力度，注重优化结构，加大对支撑结构调整的基础设施和装备建设、自主创新能力、生态环保、民生事业的投资。今年安排重点建设项目110项，完成投资359亿元，其中基础设施工程完成投资167亿元，产业项目完成投资131亿元。市政府投资计划安排162亿元，其中交通和市政基础设施投资81亿元。落实民间投资准入“负面清单”，积极鼓励民间资本发起设立创业投资基金、股权投资基金和产业投资基金，探索政府和社会资金合作模式，推动21个面向民间投资招标的项目落地建设，释放民间投资活力。实施技术改造滚动计划，发挥财政资金杠杆作用，支持企业通过技改提高装备技术水平，争取用三年时间，实现一半以上原有工业企业与行业先进对标，完成新一轮技术改造升级。今年力争完成技改投资70亿元以上。

　　大力培育消费新热点。不断催生消费新业态、新模式和新热点，争取港澳游艇自驾游、72小时免签进入横琴等政策获批，推动横琴长隆二期、海泉湾二期、桂山岛游艇度假中心一期等项目建设，打造珠港澳“一程多站”跨境旅游、万山群岛游、乡村文化和生态游等主题旅游路线。办好珠海WTA超级精英赛、第二届中国国际马戏节、首届莫扎特国际青少年音乐比赛等活动。培育若干品牌会展，扩大珠海国际游艇展等会展影响。推进4G网络建设和服务推广，发展多样化信息增值消费服务、新兴数字教育和文化娱乐消费。推广电子商务应用，支持电商企业发展，促进传统行业开展电子商务，发展移动电子商务、旅游电子商务、农产品电子商务、社区电子商务等新业态。开展家政服务体系建设试点。推动沃尔玛、中邦等商业综合体动工建设。

　　力促进出口高效益发展。支持企业扩大进出口规模，优化出口结构，促进自主知识产权、自有品牌和高新技术产品出口。实施积极的进口促进战略，鼓励企业加强先进技术、关键设备、能源资源产品和稀缺资源性产品进口。继续申报高栏港综合保税区、空港保税物流园区。建设保税区进口商品交易中心，推动保税区国际商贸服务平台建设。支持珠澳跨境工业区从以工业为主向以商贸服务业为主转型，争取设立离岸免税区，实行“一线放开、二线管住”新模式。推进珠港澳物流合作园首期通关中心项目建设。推动外贸转型升级示范基地建设，扶持研发设计、试验检测、国际营销等公共服务平台。发展外贸新业态，培育外贸综合服务企业，支持企业打造专业性综合服务平台。建立跨境贸易电子商务监管体系和服务体系，培育和引进一批企业开展跨境电子商务。建设服务贸易示范园区，拓展生物医药、文化创意、研发设计、知识产权等服务外包新领域。完善电子口岸功能，提升“三个一”通关模式运行效率，探索“单一窗口”试点工作。

　　促进实体经济发展壮大。继续培育大型骨干企业，力争主营业务收入超100亿元企业达到10家。支持“三高一特”重点企业发展，培育一批细分领域的“单打冠军”。落实民营企业“三项壮企行动”、“四项环境工程”，促进民营和中小微企业发展壮大。落实“营改增”等结构性减税政策，清理规范涉企经营性收费，减免企业应缴堤围保护费。

　　（五）大力建设国际宜居城市。

　　加强生态建设和环境保护。进一步巩固国家生态市建设成果，积极创建生态文明示范市、国家生态园林城市、国家森林城市、国家节水型城市，香洲、金湾、斗门三个区加快建成国家生态文明示范区，创建高栏港省级绿色升级示范工业园区。大力实施“美丽珠海”专项行动，全面提升城市生态品质。推进林荫道路、林荫停车场建设，引导开展立体绿化，建成绿道80公里、生态景观林带57.3公里、碳汇林1.65万亩。全面推进凤凰山森林公园等大型公园建设，新建和改造一批城市公园。基本完成裸露山体整治复绿工作，有效保护山体生态。加快完成前山河流域内污水处理工程建设和前山河流域环境综合提升工程。建成拱北污水处理厂四期工程，推进西部中信生态环保产业园一期、西坑尾垃圾填埋场生态修复、西部污泥处置中心、前山污水处理厂一期等项目。推进厨余垃圾收运处置一体化。开展一星级绿色建筑评价标识管理。开展全国水生态文明建设试点，打造“一山三河百村落、两带三园百海岛”的水生态文明总体布局。完善饮用水源保护扶持政策。启动农业发展用地、水资源保护区安全科学监测工作。加强大气污染防治，推进结构减排，实行主要污染物新增排放量等量替代或倍量替代。发展清洁生产、绿色低碳技术和循环经济。完成“黄标车”淘汰任务。探索环境执法新模式，完善公民表达环境诉求机制。促进区域环境一体化，构建生态环境管理的全方位平台。

　　推进城市优化发展。强化规划引领，深入推进“五规融合”。严守生态控制线，保护山脊线、海岸线、城市天际线。全面拉开西部生态新城建设框架，制定生态新城建设指标体系，抓紧完成土地清理整合，加快推进市政基础设施和公共服务配套建设，形成生态景观格局和主干路网格局。推进情侣路“一带九湾”建设，重点完成香炉湾、凤凰湾和唐家湾沙滩修复工程。整体打造九洲大道动感活力轴和迎宾大道绿色景观轴，重点建设圆明新园市民文化广场和佳能旧厂文化创意综合体。加快推进凤凰山旅游小镇和上冲TOD小镇建设。加快城市更新，综合改造城市之心、拱北商旅中心、九洲商贸中心以及香洲港、九洲港、洪湾渔港等地区，推进21个旧工业区和13条旧村改造项目。继续建设和改造一批农贸市场。全面推进城乡内涝整治工作。加快完善能源基础设施，发展清洁能源，推进桂山海上风电场、500千伏加林输变电工程、港珠澳大桥口岸人工岛配套供电设施等项目建设。

　　大力创建幸福村居。实现“三年大变化、五年上台阶”，成为全省示范、全国先进。继续做好“一村一品”、“一品多村”产业规划发展工作，拓展现有优势产业对周边村居的覆盖带动面，促进农村集体经济增长、农户增收。加快推进珠海国家农业科技园区、台湾农民创业园建设，示范带动全市发展都市型现代生态农业。整合乡村旅游资源，连片打造莲洲“十里风光”等乡村休闲风情带，发展生态休闲农业、乡村旅游等特色产业。扶持联户经营、专业大户和家庭农场，发展多种形式的农民新型合作组织。鼓励生产型、加工型企业延伸产业链，发展农产品家庭配送等新兴产业。大力实施村居“四整治一美化”工程。推进农村基层治理法治化建设，增强村居自治能力。建立健全警务联系村务、优秀民警驻点联系村居工作机制。

　　完善综合交通体系。同步推进港口、机场、轨道交通、高速公路、城市道路等基础设施建设，密切城市区域组团联系，改善市民出行条件。配合加快港珠澳大桥及连接线和口岸人工岛建设，加快推进金琴高速、城轨延长线等项目建设，尽快开工建设金海大桥、香海大桥、洪鹤大桥、白石桥等项目。推动广佛江珠城轨尽快动工。启动西部城区一批重点交通改扩建工程。完善港口集疏运系统，推进15万吨级主航道、疏港铁路专用线、综合物流信息平台等项目建设。加快珠海机场总体规划修编和第二跑道、通用机场规划论证报批，推动设立机场国际口岸。加快推进粤澳新通道、港珠澳大桥珠海口岸等口岸项目建设。推进珠海水域的口岸开放工作。综合治理城市交通拥堵，有效减少城市交通易堵点。加大公共交通投放力度，建设连通东西部快速公交系统和有轨电车1号线二期。加快停车场（楼）建设。建成一批人行过街设施。打通宝翠桥等珠中跨界道路。发展绿色交通，倡导绿色低碳出行。推广建设公共自行车租赁系统。

　　打造智慧城市。推动物联网、云计算、大数据等新一代信息技术创新应用，实现与城市经济社会发展深度融合。完善信息基础设施，加快光网总体建设，基本建成全覆盖的城市无线宽带网络，“政府WIFI通”覆盖重要公共服务场所，光纤入户率达到45%以上。加大公共数据的开放程度。加快推进交通运输、城市管理、电子政务、社会服务等智慧应用项目，建设云计算中心一期和智慧社区示范项目，常住人口市民卡覆盖率超过80%，市民个人网页提供服务比例超过60%。建成区完成数字城管地下管线全覆盖，优化数字城管运行机制。

　　（六）创新和加强社会建设。

　　着力解决困难群众生活问题。更加注重将民生政策更多地向困难群众、困难地区倾斜，多做雪中送炭的工作。继续加大底线民生补助力度，将城乡低保补差、五保供养、残疾人生活津贴、孤儿供养和城乡居民基本养老保险基础养老金的标准提高到全省前列水平。全面建立临时救助制度和疾病应急救助制度，逐步建立以经济困难、高龄、失能老年人为重点的老年福利制度。

　　推进教育现代化建设。创建省推进教育现代化先进市。推进教育领域综合改革，理顺教育管理体制。加快义务教育均衡优质标准化发展。加大教育资源向西部地区和农村、海岛地区倾斜，完善教师交流和支教制度。积极实施教育信息化标准工程，扩大优质教育资源覆盖面。开展特色学校建设。加快西部生态新城学校建设。推进普惠性幼儿园建设，落实小区配套幼儿园移交政策，建成10所镇中心幼儿园，全市规范化幼儿园比例达到90%。改进第四中学西藏班教学生活设施。提升高等教育质量，发展研究生教育，推动优势学科、重点实验室、协同创新中心建设。推进职业教育校企合作、产教融合。推进民办教育强市建设。重视终身教育，推动老年大学进社区。

　　加快发展卫生计生事业。深入创建健康城市，全面提升医疗卫生服务水平。推进公立医院改革，取消药品和医用耗材加成，提高医疗技术服务价格，完善政府补偿机制。发展社区卫生服务事业，开展家庭健康服务，深入探索家庭医生式团队服务新模式。发展多层次的医疗服务，扶持和促进中医药事业发展。推进市中西医结合医院、市第二中医院等中医医院（机构）建设。加快市慢性病防治中心、市妇幼保健院二期等项目建设，建成市人民医院北区项目。加强公共卫生服务体系建设，有效预防控制疾病。坚持落实计划生育基本国策，促进人口长期均衡发展。加快完善覆盖全市的多样化养老服务体系。因地制宜规划发展养老相关产业。

　　繁荣发展文体事业。构建现代公共文化服务体系，落实市、区、镇、村文化场馆免费开放，数字农家书屋全市覆盖率达到80%。全面完成珠海大剧院主体建设，市博物馆和城市规划展览馆对外开放。推动群众体育和竞技体育同步发展，举办系列群众性文体活动。继续办好沙滩音乐节、国际马拉松、国际情侣双人自行车赛等品牌活动。建立历史文化保护区和历史建筑保护名录，加强历史文化资源保护利用。推进留学博物馆、东方文化创意产业园、南方影视文化产业基地、市工人文化宫等项目建设。

　　提高社会治理水平。加大重点领域和重大社会矛盾化解力度，健全社会矛盾预防化解机制，努力实现由化解向治理、由个案解决向政策制度性消化转变，强化法律在维护社会秩序中的权威。深入推进“平安珠海”建设，完善立体化社会治安防控体系，加强反恐制暴工作，强化治安重点地区和突出犯罪活动的集中打击整治。推进拱北口岸地区等重点区域综合治理。严厉打击侵权假冒违法行为。深化信访制度改革，依法推动诉访分离。扩大法律援助覆盖面，实现应援尽援。全面加强公安工作，争取推进人民警察管理制度改革试点，加快正规化警队建设，争创全国先进公安机关。健全安全生产“党政同责、一岗双责、齐抓共管”责任体系，完善“一体系三平台”隐患排查治理体系，加强重点地区、重点企业、重点部位安全防范，深入开展打非治违专项行动，督促企业落实主体责任，加快创建国家安全发展示范城市。全面提升食品药品安全监管水平，强化基层监管力量，加强规范化管理和执法规范化建设，积极创建食品安全示范市。切实做好国防建设、国家安全、双拥优抚、民族宗教等工作。

　　（七）全心全意办好一批民生实事。

　　一是提高民生保障水平。提高城乡居民基本医疗保险财政补助水平、孤儿供养标准、困难群众医疗救助标准，降低救助门槛，开展重特大疾病医疗救助。出台残疾人医疗保障办法，提高残疾人医疗保障水平。

　　二是完善特殊教育基础设施。发展以职业教育为主的残疾人高中阶段教育，提高非义务教育阶段残疾人接受教育的比例。加快重度残疾儿童教养学校建设，满足重度残疾儿童少年的养护、康复、教育需求。开展残疾儿童康复工作。斗门区特殊教育学校投入使用。

　　三是加强医疗卫生服务。人均基本公共卫生服务经费财政补助标准提高到45元/人。推动区域医疗一卡通项目。继续推进农村妇女“两癌”免费检查。

　　四是扶持就业创业。年内城镇新增就业4.5万人，失业人员再就业1.2万人，农村劳动力转移就业2000人，就业困难人员就业2000人；新增3家市级创业孵化基地，促进创业2800人。

　　五是促进城市公共交通发展。完成一批市政道路改造工程。60周岁以上外地户籍老年人享受免费乘坐本市公共汽车待遇。优化公交线网，提升西部公交覆盖率，延长公交运营时间。规划建设一批环保电动汽车充电桩。

　　六是提升公共文化体育服务。组织开展多种形式文化活动。完善基层文化体育基础设施，按“5+2”标准新建100个村（居）文化中心，完成47个社区文体公园建设。

　　七是加大安居工程建设力度。基本建成南屏沁园1556套公租房建设项目。新开工1000套公租房项目。新开工1600套沥溪、福溪旧村改造回迁安置房项目。

　　八是优化城市生态环境。打造一批生态样板工程。建设竹洲水乡国家级水利风景区和水生态文明建设展厅。建成空气质量预报预警系统，发布生态环境指数。

　　九是改善村居生活环境。加快农村基础设施建设，农村生活污水处理覆盖到全部行政村。

　　十是加强公共法律服务。建立区、镇（街）公共法律服务中心，村（居）公共法律服务工作站。为符合条件的困难群众提供法律援助，向城乡居民提供公益、专业、均等、便民、“一站式”的基本公共法律服务。

　　（八）加快建设法治政府。

　　建立健全法制政府建设长效机制，全面推进依法行政，确保各级政府和部门在党的领导下、在法治的轨道上开展工作。

　　加强和改进政府立法。推动出台《法治政府建设条例》。健全政府立法程序，完善政府立法工作制度。完善规章立项制度，科学合理编制政府立法计划，统筹推进重点领域立法。完善规章起草制度，提高政府立法质量。完善立法项目征集和论证制度，健全公众参与立法机制，制定《政府立法公众参与办法》。落实规范性文件合法性审查制度、有效期制度、制定主体公告制度和政策解读机制。

　　提升依法治理能力。依法履行政府职责，完善法治政府制度建设，推进机构、职能、权限、程序、责任法定化。推进科学民主决策，建立健全重大行政决策程序制度，将公众参与、专家论证、风险评估、合法性审查、集体讨论决定纳入重大行政决策法定程序，制定相应细化方案或措施。推进严格规范公正文明执法，重点规范行政处罚、行政强制、行政检查等执法行为。严格执行重大执法决定法制审核和备案制度，建立健全行政执法公示制度。加大食品药品、安全生产、环境保护、劳动保障、整治违法建筑和违法用地、市容环境卫生等关系群众切身利益的重点领域执法力度，依法惩处各类违法行为。加强行政执法信息化建设和信息共享，健全行政执法联动协作机制。完善市区两级政府行政执法管理。实施行政执法和刑事司法衔接工作条例，促进严格执法和公正司法。创新法治人才培养机制，提高法治工作队伍建设水平。推进法治宣传教育方式创新，重点加强对各级领导干部尊法学法守法用法的教育培训，完成“六五”普法任务，建设珠海特色法治文化，增强全民法治意识。

　　大力推进廉政建设。强化对行政权力的制约监督，自觉接受人大、政协监督，认真落实市人大及其常委会各项决议决定，扎实办理人大议案。加强与市政协联系并通报工作，推进协商民主广泛多层制度化发展。广泛听取各民主党派、工商联、无党派人士和工青妇等人民团体意见。加强政府内部层级监督，健全行政复议案件审理机制。强化审计监督机制，保障依法独立行使审计监督权，对公共资金、国有资产、国有资源和领导干部履行经济责任情况实行审计全覆盖。加强政务公开，推进预决算信息公开，实现市本级部门决算及“三公”经费决算100%公开。加强信息公开平台建设，推进部门责任白皮书网上发布。强化党风廉政建设主体责任，深入推进政府反腐倡廉工作。推进作风建设常态化、制度化，严格落实中央“八项规定”，抓好教育实践活动整改落实，进一步转变政风。严格控制政府性楼堂馆所、财政供养人员以及“三公”经费等一般性支出，厉行节约。加强反腐倡廉基础制度建设。加强行政监察，落实行政问责制。强化责任追究，实行 “一案双查”。严肃查办发生在重点领域、关键环节和群众身边的腐败案件。坚决整治损害群众切身利益的不正之风。

　　各位代表！实干开创事业，爱拼成就梦想。让我们在新的一年里，勇于担当、奋发有为、乘势而上，为实现“共建美丽城市、共享美好生活”的城市理想而不懈奋斗！
